# Potentially Life-Threatening Phosphate Diabetes Induced by Ferric Carboxymaltose Injection: A Case Report and Review of the Literature

**DOI:** 10.1155/2014/843689

**Published:** 2014-11-13

**Authors:** Xavier Vandemergel, Frédéric Vandergheynst

**Affiliations:** ^1^Department of General Internal Medicine, Centres Hospitaliers Jolimont, 1400 Nivelles, Belgium; ^2^Department of General Internal Medicine, Hopital Erasme, 1070 Bruxelles, Belgium

## Abstract

We report the case of a 45-year-old female patient who developed phosphate diabetes after administration of ferric carboxymaltose. Ten days after the second dose, she complained of intense fatigue and blood analysis showed a phosphate plasma level of 0.93 mg/dL with phosphate excretion rate of 23%. She received phosphate supplementation which resulted in phosphate clearance improvement which persisted for two months. We reviewed other cases described in the literature and would draw attention to this rare but potentially life-threatening side effect.

## 1. Introduction 

Phosphate plays a key role in various biological processes. In recent years, new insights into the regulation of the phosphate metabolism have been obtained, including growing evidence suggesting that factors other than the parathyroid hormone (PTH) and vitamin D are involved in maintaining phosphate balance. A new class of phosphate-regulating factors, the so-called “phosphatonins,” has also been shown to play a role in phosphate-wasting diseases [1]. Among them, Fibroblast Growth Factor 23 (FGF23) is involved in various diseases, including autosomal dominant hypophosphatemic rickets/osteomalacia or tumor-induced hypophosphatemic rickets/osteomalacia [2]. True hypophosphatemia can be induced by a decrease in net intestinal absorption, increase in urinary phosphate excretion, or acute movement of extracellular phosphate into the cells. Then, the normal renal response to phosphate depletion is to increase phosphate reabsorption, leading to the virtual abolition of urinary phosphate excretion. Most of the filtered phosphate is reabsorbed in the proximal tubule through the sodium-phosphate cotransporter in the luminal membrane [3]. Regarding hypophosphatemia, phosphate diabetes is defined by a phosphate clearance >15 mL/min with a proximal tubular reabsorption rate <85%. The formula used to calculate the fractional phosphate excretion (FEPO_4_) is the same as that used for the fractional sodium excretion. FEPO_4_ should be much less than 5% (normal range: 5–20%) if the kidney responds normally and renal phosphate wasting is not the cause of hypophosphatemia. A phosphate plasma level <1 mg/dL can be fatal because it can lead to cardiac arrest and severe hypophosphatemia can lead to metabolic encephalopathy and could therefore contribute to the development of central and extrapontine myelinolysis [4–6]. Iron deficiency anemia affects 1-2% of adults [7] while iron deficiency without anemia is more prevalent, affecting up to 11% of women. In adults with iron deficiency anemia, presenting symptoms usually include weakness, headache, irritability, fatigue, and exercise intolerance. Although symptoms of iron deficiency without anemia remain puzzling, Vaucher et al. have shown that iron supplementation should be discussed in women with unexplained fatigue whose ferritin levels are less than 50 *μ*g/L [8]. Moreover, in the short-term study by Anker et al. conducted in patients with chronic heart failure and iron deficiency with or without anemia, treatment with intravenous ferric carboxymaltose (FCM) has been shown to improve symptoms, functional capacity, and quality of life [9]. Thus, treatment with iron, especially intravenous FCM preparation, is increasingly used due to its higher efficacy and decreased side effects, which are mainly gastrointestinal, compared with oral iron therapy. In 2008, the FDA has reported that hypophosphatemia was frequently found after FCM administration but only one patient was reported with a phosphate level <1 mg/dL. We describe the case of a patient with profound hypophosphatemia after FCM administration.

## 2. Case Report

**Figure 1:**
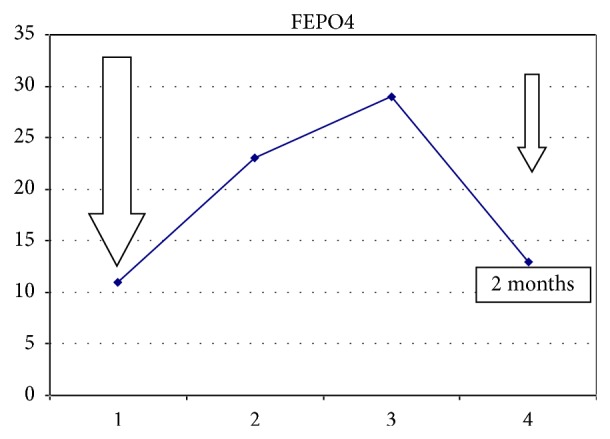
Evolution of FEPO_4_ (%) after administrations of ferric carboxymaltose.

A 45-year-old African woman had iron deficiency for 4 years caused by abundant menstruation. Her past medical history included tuberculosis in 1985 and lung lobectomy for aspergilloma five years earlier. She had 4 healthy children and did not smoke. Gastroscopy performed initially was normal. She complained of fatigue and headache. She took oral iron supplementation but developed side effects that led to treatment discontinuation. In 2011, she received two infusions of iron sucrose (total dose: 200 mg) without any impact on the phosphate level (2.7 mg/dL after the second infusion). In 2012, laboratory findings were hemoglobin level (Hb) of 9.9 gr/L, serum ferritin of 6 *μ*g/L (normal range: 13–150), and serum iron saturation of 6.7% (normal range: 15–50). She received 3 additional infusions of iron sucrose (total dose: 300 mg) which slightly reduced the phosphate level (2.1 mg/dL) but without symptoms. In 2014, her Hb was at 9.8 gr/dL with serum iron saturation at 6% and serum ferritin at 13 *μ*g/L, and the plasma phosphate level before injection of FCM was of 2.5 mg/dL with a FEPO_4_ of 11%. This moderate elevation in phosphate clearance was probably due to 25-hydroxyvitamin D (25(OH)D3) deficiency and secondary hyperparathyroidism (PTH: 147.2 ng/L, normal range: 15–65; 25(OH)D3: 9 ng/mL, normal range: >30). She then received two injections of FCM (Injectafer, Vifor Pharma) (one-week interval, total dose: 1000 mg). Ten days after the second infusion, she complained of intense fatigue. Blood analysis showed a phosphate level at 0.93 mg/dL with FEPO_4_ at 23% confirming the diagnosis of phosphate diabetes. She received oral phosphate supplementation which improved fatigue and at, one month, the phosphate level increased to 1.2 mg/dL with FEPO_4_ at 29%. Two months after the first administration, the phosphate level returned to normal value at 2.34 mg/dL with FEPO_4_ at 13%.  Table 1 summarizes the evolution of laboratory results before and after FCM administration and the evolution of fractional phosphate excretion over time is presented in Figure 1.

## 3. Discussion

Phosphate disorders are frequently found in outpatient clinic and hospital departments. In the hospital setting, Halevy and Bulvik [10] have shown that severe hypophosphatemia (serum phosphate level less than or equal to 0.48 mmol/L or 1.5 mg/dL) predominantly developed in postoperative care but medications known to precipitate hypophosphatemia were a causative factor in most patients. In their study, the mortality rate was of 30% in patients with serum phosphate concentration less than or equal to 0.32 mmol/L (or 1.0 mg/dL). The cause of death and its temporal association with the lowest phosphate concentration observed indicate that severe hypophosphatemia could be a factor contributing to mortality.

Some studies of intravenous FCM injection have reported a decrease in serum phosphate levels. FCM-induced hypophosphatemia was usually asymptomatic and transient [11, 12]. We found six cases of profound hypophosphatemia postintravenous FCM injection in the literature (Table 2) [13–15]. All were women aged between 24 and 47 years. Two patients had had kidney transplantation, one had history of anorexic/bulimic disorder, one had systemic lupus erythematosus with antiphospholipid syndrome, and one had undergone laparoscopic Roux-en-Y gastric bypass two years earlier. The most frequent symptom was persistent weakness in four cases which was asymptomatic in three cases. Nausea was present in one case. 25(OH)D3 deficiency with hyperparathyroidism was not always present. Phosphatemia after injection ranged between 0.5 mg/dL and 0.93 mg/dL and FEPO_4_ between 9 and 59%. Hypophosphatemia usually appeared one week after the first dose of FCM. In one case, the patient developed vertigo, diarrhea, and tingling in both hands. As in our case, the resolution of the hypophosphatemia often needed several weeks despite phosphate supplementation and was difficult to achieve. In one case, the hypophosphatemia persisted for several months despite supplementation. In one case, because of the persistent anemia, a single infusion of iron sucrose (100 mg Venofer, Vifor) was subsequently administered and resulted in profound hypophosphatemia (1 mg/dL).

Phosphate homeostasis is maintained via the bone-kidney endocrine axis which is mainly regulated by PTH, vitamin D, and FGF23. In addition, PTH and FGF23, two phosphaturic hormones, also regulate the renal phosphate excretion [16]. In 4 cases, hyperparathyroidism was present but it seems unlikely that the degree of hypovitaminosis D with only a slight upregulation of PTH can explain the deep hypophosphatemia by itself. Moreover, in our case, the PTH level did not change before and after FCM injection. Profound hypophosphoremia appeared in one patient with malnutrition [15], after renal transplantation in two patients receiving tacrolimus [13, 14], and two years after a laparoscopic Roux-en-Y gastric bypass in one case. However as in our case, it can occur without malnutrition or kidney diseases. Predominance of women is probably due to the etiology of anemia which was often due to abundant menstruation. The dose of FCM does not seem to be correlated with the importance of hypophosphatemia.

FGF23 has been proposed to be involved in the occurrence of hypophosphatemia after iron administration [17, 18]. FGF23 is a phosphatonin secreted by osteocytes and osteoblasts leading to phosphate wasting through inhibition of Na^+^-dependent phosphate cotransporters in the renal proximal tubules [19]. Wolf et al. [18] have tested the association of iron deficiency anemia with C-terminal FGF23 (cFGF23) and intact FGF23 (iFGF23) levels in 55 women with history of heavy uterine bleeding and assessed the longitudinal biological response over 35 days to equivalent doses of randomly-assigned, intravenous elemental iron as FCM or iron dextran. They have shown that iron deficiency was associated with markedly elevated cFGF23 levels but normal iFGF23 levels at baseline. After iron administration, cFGF23 levels dropped by 80% in both groups (dextran and carboxymaltose) whereas iFGF23 increased only in the FCM group. Reduced serum phosphate was accompanied by increased fractional phosphate excretion, decreased calcitriol levels, and increased PTH levels. The authors have suggested that intravenous iron could lower cFGF23 in humans by reducing FGF23 transcription as in mice whereas carbohydrate moieties in certain iron preparations could simultaneously inhibit FGF23 degradation in osteocytes leading to transient increases in FGF23 and reduced serum phosphate. Schouten et al. [17] have shown that phosphate levels were decreased in 8 patients receiving iron intravenous administration from 3.4 ± 0.6 mg/dL to 1.8 ± 0.6 mg/dL and the tubular reabsorption rate was reduced from 90% ± 4.8% to 68% ± 13% after one week. More interestingly, they have also shown that 1,25(OH)D3 was rapidly and dramatically inhibited while the PTH level was significantly increased at week 3 as well as the iFGF23 level (from 43.5 pg/mL (38–49) to 177 pg/mL (199–260)) which correlated with the serum phosphate level.

In nondialysis chronic kidney disease (CKD) patients, however, cFGF23 dropped significantly 3 weeks after FCM injection [20] without changes in 1,25(OH)D3. Thus, it seems that the presence or absence of CKD could result in interindividual differences. A study comparing the occurrence of hypophosphatemia in women with iron deficiency secondary to heavy uterine bleeding treated with intravenous FCM versus iron dextran is ongoing and FCM could have a greater efficacy on phosphate metabolism. However, osteomalacia has also been described in patients treated with saccharated ferric oxide [21, 22]. No case of osteomalacia was identified in the literature using iron dextran and Sanai et al. [23] have shown in an animal model that the tubular phosphorus reabsorption was greater in patients treated with iron dextran than in those treated with saccharated ferric oxide and in untreated patients.

In conclusion, we report the case of a woman with reversible phosphate diabetes and profound hypophosphatemia after FCM administration. Long-term monitoring of phosphate level is mandatory during FCM treatment and physicians must be aware of this potential side effect. We retrospectively reviewed all FCM injections administrated in our hospitals over 3 years. Among the 50 patients who had received FCM, three had developed profound hypophosphatemia after injection (<1 mg/dL). It should be noted that the phosphate level had been measured only in 50% of patients after injection. It can be assumed that FGF23 plays a role and iron anemia, which increases the FGF23 level, could be another explanation of this potentially lethal side effect. More studies are needed to assess the impact of high-dose FCM intravenous injection on phosphate metabolism and to balance its benefit/risk ratio.

## Figures and Tables

**Table 1:** Evolution of laboratory results.

	Initial values	10 days after the second FCM administration
Ca^++^ (mmol/L)	2.74	2.61
Cr (mg/dL)	0.5	0.5
P pl (mg/dL)	2.5	0.93
PTH (ng/L)	147	165
25(OH)D3 (ng/mL)	9	14
Fractional excretion of phosphate (%)	11	23

PTH: parathyroid hormone; 25(OH)D3: 25-hydroxyvitamin D3.

**Table 2:** Biological parameters of patients reported in the literature.

	Blazevic et al. [13]				Mani et al. [14]	Fierz et al. [15]	This case
Doses of Ferric carboxymaltose (gr)	1	3	2	3	1.5	1	1
P04 before administration (mg/dL)	?	?	?	?	?	?	2.5
Nadir P04 pl (mg/dL)	0.78	1	0.68	0.8	0.5	0.56	0.93
FEPO_4_ before administration (%)	?	?	?	?	?	?	11
FEPO_4_ after administration (%)	59	12.4	23	28	9	77	23
PTH	Elevation	normal range	Elevation, 16.7 pmol/L	?	?	Undetectable one week after first dose	Moderate elevation before administration. No change
Vitamin D	Lower	Normal range	28 nmol/L	?	36 (48–60)	Normal range	9 ng/mL
FGF23 (normal <125 Ru/mL)	202			119 at six weeks			
